# Convergence of developmental mutants into a single tomato model system: 'Micro-Tom' as an effective toolkit for plant development research

**DOI:** 10.1186/1746-4811-7-18

**Published:** 2011-06-29

**Authors:** Rogério F Carvalho, Marcelo L Campos, Lilian E Pino, Simone L Crestana, Agustin Zsögön, Joni E Lima, Vagner A Benedito, Lázaro EP Peres

**Affiliations:** 1 Laboratory of Hormonal Control of Plant Development, Department of Biological Sciences (LCB), Escola Superior de Agricultura "Luiz de Queiroz" (ESALQ), Universidade de São Paulo (USP) - Av. Pádua Dias, 11, CP 09, CEP 13418-900 Piracicaba - SP, Brazil; 2 Center for Nuclear Energy in Agriculture (CENA), USP, Av. Centenário, 303, CEP 13400-970 Piracicaba, SP, Brazil; 3 Genetics and Developmental Biology Program, Plant and Soil Sciences Division, West Virginia University, 2090 Agricultural Sciences Building, Morgantown, WV 26506, USA

**Keywords:** hormonal mutants, *Solanum lycopersicum*, model organism, photomorphogenesis, plant development

## Abstract

**Background:**

The tomato (*Solanum lycopersicum *L.) plant is both an economically important food crop and an ideal dicot model to investigate various physiological phenomena not possible in *Arabidopsis thaliana*. Due to the great diversity of tomato cultivars used by the research community, it is often difficult to reliably compare phenotypes. The lack of tomato developmental mutants in a single genetic background prevents the stacking of mutations to facilitate analysis of double and multiple mutants, often required for elucidating developmental pathways.

**Results:**

We took advantage of the small size and rapid life cycle of the tomato cultivar Micro-Tom (MT) to create near-isogenic lines (NILs) by introgressing a suite of hormonal and photomorphogenetic mutations (altered sensitivity or endogenous levels of auxin, ethylene, abscisic acid, gibberellin, brassinosteroid, and light response) into this genetic background. To demonstrate the usefulness of this collection, we compared developmental traits between the produced NILs. All expected mutant phenotypes were expressed in the NILs. We also created NILs harboring the wild type alleles for *dwarf*, *self-pruning *and *uniform fruit*, which are mutations characteristic of MT. This amplified both the applications of the mutant collection presented here and of MT as a genetic model system.

**Conclusions:**

The community resource presented here is a useful toolkit for plant research, particularly for future studies in plant development, which will require the simultaneous observation of the effect of various hormones, signaling pathways and crosstalk.

## Background

In addition to its worldwide cultivation and economic importance, tomato (*Solanum lycopersicum *L.) has several characteristics that make it a convenient model plant species, such as a relatively compact genome (950 Mb) combined with a marker-saturated genetic linkage map [1], rich germplasm collections (Tomato Genetics Resource Center) [2] and highly efficient transformation protocols [3]. The pre-released annotated genome sequence [1] appears set to establish tomato as a prominent model system for research into plant genetics and physiology. The tomato is poised to become an alternative model plant to *Arabidopsis thaliana *due to its diverse developmental traits not found in *Arabidopsis*. These traits include: the photoperiod-independent sympodial flowering and the formation of fleshy climacteric fruits, compound leaves, mycorrhizal roots and glandular trichomes.

The convenient small size and amenability to large-scale cultivation of *Arabidopsis *are also found in tomato cv. Micro-Tom (MT) [4]. MT was initially created for ornamental purposes [5], but its rapid life cycle and high-throughput capabilities indicate that MT is a candidate cultivar as tomato's model system [6]. Regardless of the presence of mutations that cause the MT's dwarf size, it has been proven to be suitable as a standard genotype in tomato research [see 3, 7], including the study of novel hormonal interactions [8,9].

Mutants are the most classical and probably the most reliable genetic tool for accessing biological information in a living organism. The plethora of mutants available in tomato is another advantageous characteristic of this model plant [10]. However, as for every model organism, comparative studies using tomato mutants tend to be limited by the difference in genetic backgrounds, given that the same gene function can have diverse effects depending on epistatic interactions with other genes [11,12]. In tomato, most of the known mutations are distributed in various genetic backgrounds including heirlooms, hybrids, and wild species [2].

By using a series of backcrosses, we introduce a rich collection of tomato hormonal and phytochrome mutants introgressed into a unique background, the MT cultivar. Merging the benefits of MT and the vast range of already well-characterized mutations, this collection encompasses a powerful and ready-to-use toolkit for studying plant genetics and physiology, allowing comparisons between different mutants without the issue of background noise. To emphasize the potential of this toolkit, we also present novel observations made with the mutants, and review some published data in light of the present collection.

## Results

### The developmental mutant collection in the tomato cv. Micro-Tom

Reliable comparative studies of specific genes mutated in pathways leading to hormone deficiency or insensitivity, as well as response to light, can only be performed in a uniform genetic background [11]. Because of its benefits as a plant model with small size and rapid life cycle [4,6], we chose the MT cultivar as the recurrent parental to introgress available hormonal (Table 1) and photomorphogenetic (Table 2) mutations. The introgression consisted of a series of successive back-crosses up to BC_6 _generation, when at least 99% of the plants' genome corresponds to MT (see Additional file 1: Figure S1 for an introgression scheme).

**Table 1:** Hormonal mutations introgressed into cv. Micro-Tom

Mutant	**Hormonal class**^**a**^	Effect/Gene function	Origin	Reference
*diageotropica (dgt)*	Auxin	Low sensitivity. Defect in a cyclophilin biosynthesis gene (a putative signal transduction component)	LA1529 cv. unknown	[14]
*Never ripe (Nr)*	Ethylene	Low sensitivity. Defective for an ethylene receptor	LA0162 cv. Pearson	[17]
*epinastic (epi)*	Ethylene	Ethylene overproduction. Unknown gene function	LA2089 cv. VFN8	[16]
*sitiens (sit)*	ABA	ABA deficiency. Defective for ABA-aldehyde oxidase	LA0574 cv. Rheinlands Ruhm	[26]
*flacca (flc)*	ABA	ABA deficiency. Defective for maturation of ABA-aldehyde oxidase Mo cofactor	LA0673 cv. Rheinlands Ruhm	[26]
*notabilis (not)*	ABA	ABA deficiency. Defective for NCED (carotenoid cleavage enzyme).	LA0617 cv. Lukulus	[27]
*gibberellin deficient 1 (gib1)*	GA	GA deficiency. Defective for *ent*-copalyl diphosphate synthase (CPS)	LA2893 cv. Moneymaker	[21]
*gibberellin deficient 2 (gib2)*	GA	GA deficiency. Defective for conversion of *ent*-7α-hydroxykaurenoic acid to GA_12_-aldehyde	LA2894 cv. Moneymaker	[21]
*gibberellin deficien t3 (gib3)*	GA	GA deficiency. Defective for *ent*-kaurene synthase (KS)	LA2895 cv. Moneymaker	[21]
*procera *(*pro*)	GA	Constitutive response. Contains a point mutation in a gene that converts the VHVID putative DNA-binding domain of the tomato *DELLA *gene into VHEID	LA0565 cv. Condine Red	[19]
*curl 3 (cu3)*	BR	Decreased sensitivity. Defective for BR receptor (*LeBRI1*) found in *S. pimpinellifolium*	LA2398 wild species	[24]
*dumpy (dpy)*	BR	BR deficiency. Probably defective in the conversion of 6-deoxocatasterone to 6-deoxoteasterone	LA0811 cv. unknown	[23]

^a ^ABA = abscisic acid; GA = gibberellin; BR = brassinosteroid.

**Table 2:** Photomorphogenic mutations introgressed into cv. Micro-Tom

Mutants	Mutation	Gene function	Origin	Reference
*aurea (au)*	Deficiency in phytochrome chromophore biosynthesis	Defective for the phytochromobilin synthase gene	LA3280 cv. Ailsa Craig	[31]
*yellow green 2 (yg2)*	Deficiency in phytochrome chromophore biosynthesis	Probably defective for the heme oxygenase gene	LA2514 cv. unknown	[32]
*high pigment 1 (hp1)*	Increased response to light	Defective for a gene homologous to *DDB1A *of *Arabidopsis*, which codes a protein interacting with DET1 (HP2), a repressor of photomorphogenesis	LA3004 cv. Rutgers	[35]
*high pigment 2 (hp2)*	Increased response to light	Defective for a gene homologous to *DET1 *of *Arabidopsis*, a negative repressor of photomorphogenesis	LA2451 cv. Manapal	[36]
*atroviolacea (atv)*	Increased response to light	Natural variation from *S. cheesmaniae*, probably a non-functional allele of a negative regulator of photomorphogenesis	LA0797 Hybrid	[38]
*Intense pigment (Ip)*	Increased response to light	Natural variation from *S. chmielewskii*, probably a positive regulator of light response, whose tomato allele is non-functional	LA1563 Hybrid	[38]

**Figure 1:**
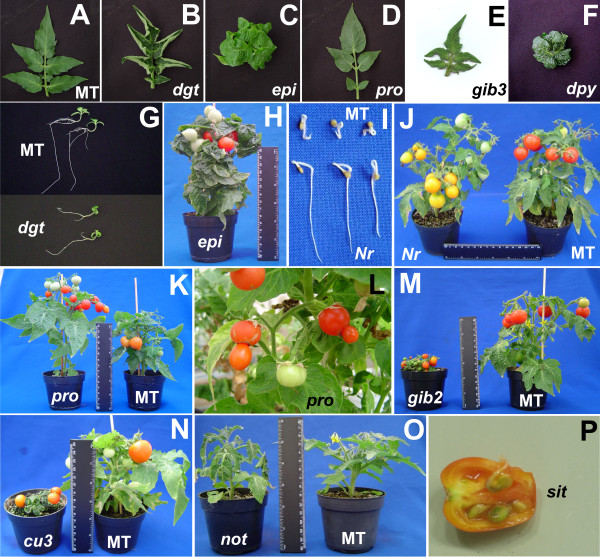
**Phenotype of hormone mutants introgressed into cv. Micro-Tom (MT)**. Leaf phenotype of MT (A), *dgt *(B), *epi *(C), *pro *(D), *gib3 *(E) and *dpy *(F). (G) Reduced gravitropic response and lateral root formation in 10-day old seedlings of the auxin mutant *dgt *when compared to MT. (H) Severe epinasty of the ethylene overproducer mutant *epi *in the MT background resulting in a phenotype where stems are hardly observable. (I) When growing in 200 ppm ethrel (an ethylene-releaser), MT seedlings show short roots and hypocotyls with exaggerated hook, a phenotype not observed in the ethylene low sensitive *Nr*. (J) *Nr *also shows incomplete ripening, producing yellow fruits. (K) Phenotype of *pro *with increased stem elongation and navel fruits (L). (M and N) Phenotypes of *gib2 *(M) and *cu3 *(N), with severe plant size reduction and leaf expansion inhibition compared to MT. (O) ABA deficiency in *notabilis *leads to wilting during the hottest hours of the day. (P) Precocious germination (vivipary) in *sit *seeds within the fruit. A description of hormone alterations involved in each mutant can be found on Table 1. Ruler in (H), (J), (K), (M), (N) and (O) = 15 cm.

**Figure 2:**
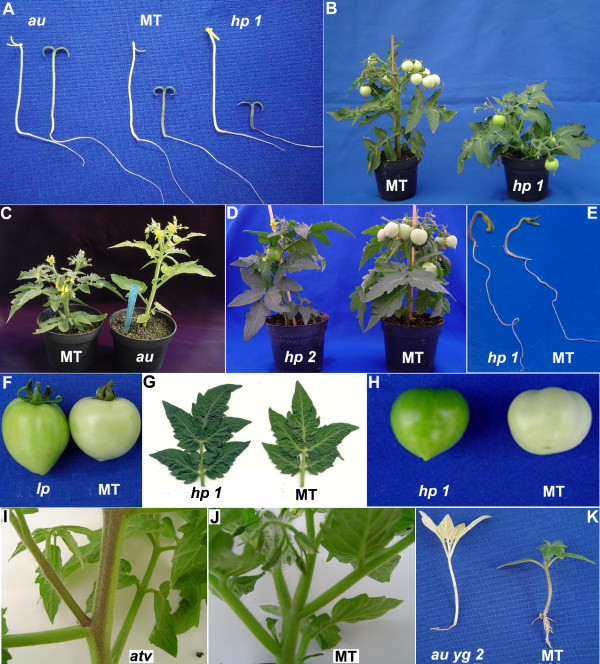
**Phenotype of photomorphogenic mutants introgressed into cv. Micro-Tom**. (A) *au*, MT and *hp1 *seedlings grown in the dark (left) or light (right). Note that in the dark, the three genotypes do not differ in hypocotyl length, however, in the light, *au *appears etiolated and *hp1 *shows higher de-etiolation than MT. (B) Reduced plant size and dark fruits in *hp1*. (C) Etiolation in *au *in the light leads to taller and chlorotic plants. The length of the pipette tip is 8 cm. (D) Anthocyanin accumulation and dark-green pigmentation in *hp2 *leaves. (E) Anthocyanin accumulation in light-grown *hp1 *hypocotyls. (F) Increased pigmentation in *Ip *fruits. (G) Increased pigmentation in *hp1 *leaves. (H) Increased pigmentation in *hp1 *fruits is stronger than in *Ip *(F). (I) Anthocyanin accumulation in *atv *stems. (J) Absence of visible anthocyanin pigmentation in MT shoots. (K) Decreased chlorophyll pigmentation in both *au *and *yg2 *makes the double mutant *au yg2 *almost albino and lethal (plants usually die before the second pair of true leaves). See Table 2 for a detailed description of gene functions of photomorphogenic mutants.

Plants were selected after each backcross based on known phenotypic characteristics of the mutants (described in the references in Table 1 and Table 2) and also the miniature and determinate growth habit of MT [13]. The description of the mutant phenotypes in their original genetic background can be found in the Tomato Genetics Resource Center (TGRC) website [2]. Some of the most conspicuous traits are shown in Figures 1 and 2 for hormonal and photomorphogenetic mutants, respectively, as proof of concept that the introgressions were successful.

MT leaves usually have five leaflets, which are rarely hyponastic or epinastic, with slightly notched leaf margins (Figure 1A). On the other hand, leaves of the reduced auxin sensitivity mutant *diageotropica *(*dgt*) [14] introgressed into the MT background present severe hyponasty (Figure 1B). The *dgt *mutation in MT also led to the characteristic altered gravitropic response and reduced number of lateral roots (Figure 1G). These phenotypes agree with the role of auxin in plant development [14] and are also observable in the original *dgt *parental line [15].

Leaf development is altered in the ethylene-overproducing mutant *epinastic *(*epi*) [16], which is thus named because of its severely epinastic (curled downward) leaves (Figure 1C). The curled leaves of *epi *in the miniature MT background resulted in a plant with barely visible stems (Figure 1H). The *epi *mutant also showed the exaggerated stem thickening and root branching (data not shown) observable in the parental plant as described by Fujino *et al*. [16].

Another ethylene mutation introgressed into MT was the reduced ethylene sensitivity *Never ripe *(*Nr*) [17]. In the MT background *Nr *shows all traits typical of an ethylene insensitive mutant [18]. The ethylene triple response phenotype (thickening and shortening of hypocotyl with pronounced apical hook) is absent in MT-*Nr *seeds germinated in the presence of ethylene (Figure 1I), as reported for the mutant in the original background by [18]. Also absent in MT-*Nr *plants are senescence and abscission of leaves and flowers, with petals and anthers remaining attached even upon fruit development (not shown). The unripe fruit phenotype is the most representative trait of this mutant; *Nr *fruits linger with an orange/yellowish color (Figure 1J). In spite of this, seeds of MT-*Nr *are as viable as MT seeds (see below).

The monogenic recessive *DELLA *mutant *procera *(*pro*) [19], originally isolated in the cultivar Condine Red, leads to a constitutive gibberellin (GA) response phenotype which includes increased height, more elongated internodes, thinner leaves and reduced leaf lobing [20]. All of these phenotypes are evident in MT-*pro *(Figures 1D, 1K). Interestingly, we also observed that MT-*pro *plants have a higher tendency to form parthenocarpic and navel fruits, compared to MT (Figure 1L).

Three other GA mutants were introgressed: *gibberellin-deficient 1*, *2 *and *3 *(*gib-1*, *gib-2 *and *gib-3*). These mutants are disrupted at different points of the GA biosynthetic pathway [21] (Table 1). They require GA to germinate, develop flowers, set fruits and produce seeds in their original background [22], and all these traits have been maintained in the MT introgressed lines. Figure 1M shows the severe dwarfism of MT-*gib2 *(*gib1 *and *gib3 *present a similar phenotype - not shown), consistent with the expected phenotype for a GA mutant. The leaflet margins of these three GA defective mutants, as opposed to the excess-GA mutant *pro *and to MT, show a very serrated lobe pattern (Figure 1E for *gib3*).

Brassinosteroid (BR) mutants are generally severely dwarfed with extremely reduced and curled leaves, as in the tomato BR defective *dumpy *(*dpy*) [23] and the tomato BR insensitive *curl3 *(*cu3*) [24] mutants. The MT BR mutants *dpy *and *cu3 *(Figure 1N) also showed reduced and curled leaves (Figure 1F), as expected. The introgression of these mutations in MT created what, to the best of our knowledge, are the smallest viable tomato lines described to date, with adult plants less than 3 cm tall. As mentioned above, *gib *mutants are also very small (Figure 1M), but they do not produce seeds without exogenous GA application. Application of a bioactive BR, such as brassinolide, attenuates the phenotype of *dpy*, but not of *cu3 *[23].

Abscisic acid (ABA) is sometimes referred to as the "stress hormone" because of its involvement in many biotic and abiotic stress responses [25]. Three mutants impaired in ABA biosynthesis were introgressed in this work: *sitiens *and *flacca *(*sit *and *flc *respectively) [26], originally from the Rheinlands Ruhm cultivar, and *notabilis *(*not*) [27] introgressed into MT from cv. Lukullus. Consistent with the role of ABA in drought stress, and as observed in the parental lines, MT ABA-deficient mutants present severe wilting when exposed to a mild drought stress, as exemplified for *not *in Figure 1O. ABA also plays an important role in seed development and primary seed dormancy [28] and the ABA-deficient mutant seeds frequently showed vivipary (Figure 1P), which is more easily observable in *sit *than in *not *or *flc*.

Light is one of the most important environmental factors conveying information on the plants' environment, and irradiance can quickly alter plant development at various instances [29]. Phytochromes are photoreversible light perception proteins involved in seed germination, seedling establishment, de-etiolation, shade avoidance, flowering, and many other processes [30]. Two phytochrome-deficient mutants were introgressed into MT: *aurea *(*au*), a tomato mutant defective in one of the last steps of the phytochrome chromophore biosynthesis pathway [31] and *yellow green 2 *(*yg2*), which is probably defective for the heme oxygenase gene [32]. Both of these mutations can be readily identified by characteristic elongated hypocotyls and paler green leaves when grown under white light (as shown for *au *in Figures 2A and 2C, respectively). The difference in height between these mutants [33] is more clearly revealed in the MT miniature background. As shown in Figure 2C for MT-*au*, these plants are taller than their MT parent. MT-*au *and MT-*yg2 *plants are also less branched than MT. Since the chromophore is common for all phytochrome types, it is presumed that *au *and *yg2 *mutants present alterations in responses controlled by both type I (*phyA*) and type II (*phyB1*, *phyB2*, *phyE*, *phyF*) phytochromes in tomato. The *au yg2 *double mutant has an additive chlorophyll deficiency (Figure 2K), confirming that both mutations are weak alleles controlling different steps in the chromophore biosynthetic pathway, as suggested by van Tuinen *et al*. [34].

The non-allelic tomato high pigment mutations *high pigment 1 *(*hp1*) [35] and *high pigment 2 *(*hp2*) [36] were introgressed into the MT cultivar from cultivars Rutgers and Manapal, respectively. These monogenic recessive mutations have an exaggerated de-etiolation photoresponse. Both *hp1 *(Figure 2A) and *hp2 *seedlings present inhibition of hypocotyl elongation and intense anthocyanin accumulation (Figure 2E) when grown under white light [37]. Also conspicuous in both *hp1 *and *hp2 *are high chlorophyll pigmentation in leaves (Figure 2G for *hp1 *and 2D for *hp2*) and fruits (Figure 2H for *hp1*, and also Figure 2C). These phenotypes are in agreement with the known function of the *HP1 *and *HP2 *genes in the light signal transduction pathway (Table 2).

Natural genetic variation for light response, the *atroviolacea *(*atv*) and *Intense pigment *(*Ip*) [38] alleles were also brought into the MT genetic background. These mutants are phenotypically similar to *hp *mutants, but map to different loci [39,40]. Derived from *Solanum cheesmaniae*, the *atv *mutant is characterized by an excess of anthocyanin in stems, leaves and fruits. Figure 2I shows the typical accumulation of this pigment in MT-*atv *stems (Figure 2J). *Ip *is a mutation originally found in the wild tomato relative *Solanum chmielewskii*, which confers darker pigmentation in unripe and ripe fruits. This characteristic phenotype is present in the MT-*Ip *(Figure 2F).

### Comparative studies made easy using the MT developmental mutant collection

A set of mutations harbored in a single genetic background allows comparative studies without having to deal with possible background-specific modifiers. To illustrate this point, we carried out some simple comparative experiments between our MT hormonal and photomorphogenetic mutants, recording several parameters during plant development, from seed germination to fruit set. The aim of these analyses was not to prove the involvement of a specific gene in a given biological process, but rather to demonstrate a general participation of a given hormone/light response in plant development and as proof of concept for the usefulness of the collection in comparative studies. These results also provide further evidence confirming that the mutations were correctly introgressed.

#### Seed germination

**Figure 3:**
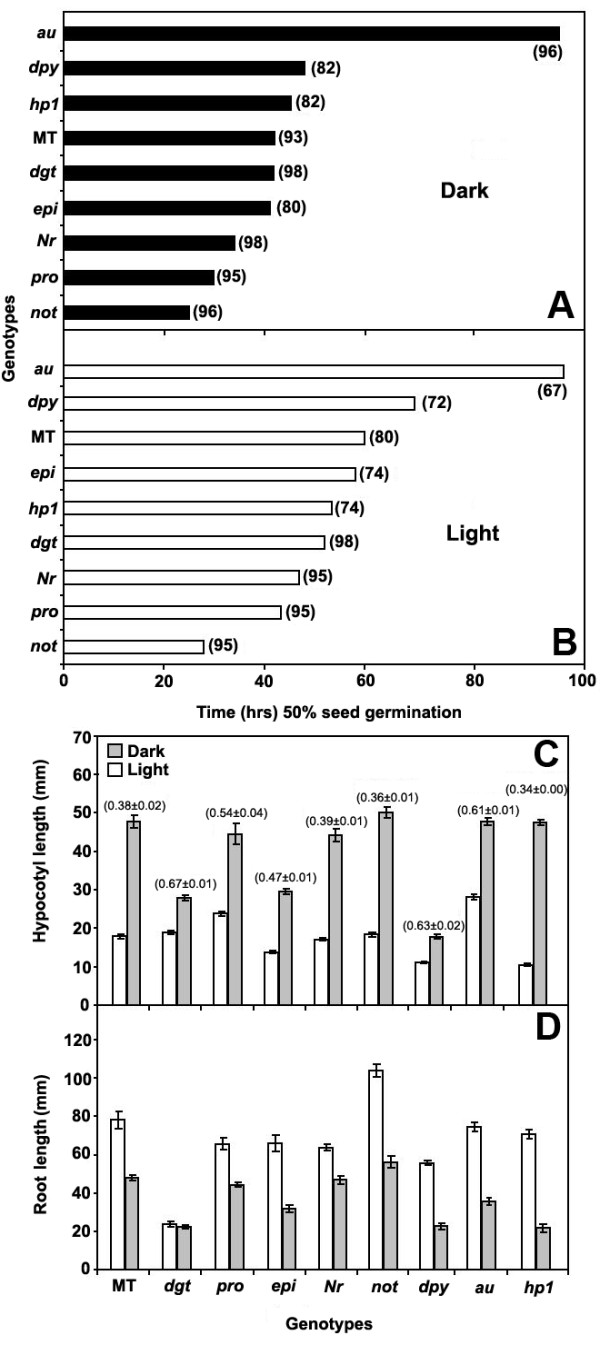
**Seed germination and seedling growth in hormone and photomorphogenic mutants**. (A-B) Time (days) to reach 50% germination of seeds in dark (A) or light (B). Final germination percentage is between brackets. (C-D) Hypocotyl and root length of light-grown (open bars) and dark-grown (closed bars) seedlings during 10 days. In (C), numbers between brackets represent the ratio between hypocotyl length in the light and dark. Vertical lines represent standard error (n = 3 × 50 for germination and n = 20 for hypocotyl and root length).

Although virtually every hormone has been suggested to have a role in seed germination [41], the classic and antagonistic hormones associated with this process are GA and ABA, inducing and repressing seed germination, respectively [42]. The *pro *GA-constitutive mutant shows a significant reduction in time required for 50% germination, both in light and dark, compared to MT (Figure 3A, 3B). A similar effect was observed in the ABA-deficient *not *mutant (Figure 3A, 3B).

Besides ABA and GA, another hormone with a fundamental role in seed germination is ethylene [41]. Early germination was observed for the partially ethylene insensitive *Nr *mutation in the MT background, a result reported previously for tomato [41], but opposite to that observed in *Arabidopsis *ethylene insensitive mutants [43,44]. The ethylene overproducer *epi*, however, showed no difference with respect to MT (Figures 3A, 3B). The role of auxin in embryogenesis is well described, but little is known about its activity during seed germination [41]. The *dgt *mutant (reduced sensitivity to auxin) showed an accelerated germination in the light but not in the dark (Figures 3A, 3B), suggesting an interaction with either phytochrome or other light receptors. The BR-deficient *dpy *had a slow germination rate, significantly more so in the light than in the dark. This is not unexpected, as BR has been reported to promote germination [45]. Finally, the MT background did not affect the previously described response on seed germination time in photomorphogenic mutants, *i.e*. *au *has a slower germination [46], and *hp1 *had no effect on germination time [47].

#### Hypocotyl elongation

Light inhibits hypocotyl elongation through a signal transduction pathway starting with photoreceptors but whose downstream components are still to be unveiled [48]. Hormones are also strong candidates to participate in this pathway [49]. The ratio between the lengths of dark- and light-grown hypocotyls can be used as a parameter to screen for mutations affecting etiolation in the dark or de-etiolation in the light. Here, hypocotyl length of either light (16 h photoperiod) or dark-grown mutants was measured 10 days after germination. The phytochrome-deficient *au *mutant presented a reduced light inhibited growth (Figures 2A and 3C), but its etiolation in dark was equivalent to that of MT (Figure 3C). On the other hand, a mutant with exaggerated phytochrome response, *hp1*, showed higher inhibition of hypocotyl elongation in the light (Figure 3C and see also Figure 2A). These results are in agreement with previous reports for these mutants [34,47], and demonstrate that the MT background is not epistatically affecting the phenotype conferred by such mutations.

The hormonal mutants *dgt*, *dpy*, *pro*, and *epi *showed high ratios between hypocotyl lengths under light vs. dark, when compared to MT. The high value for *pro *can be attributed to the mutant having significant growth in the light (Figure 3C). On the other hand, the reduced difference between light and dark for *dgt*, *epi*, and *dpy *is probably due to a higher inhibition of elongation in the dark (Figure 3C). The requirement of auxin (*dgt*) for elongation in the dark and the positive effect of GA (*pro*) under light, but not in the dark, have already been suggested for tomato and other model species [50,51]. Furthermore, the response of *epi *is consistent with ethylene's known inhibitory effect on hypocotyl elongation [52,53]. Although the BR-deficient mutant *dpy *presented a much reduced hypocotyl length in both conditions, the inhibition was higher in the dark (Figure 3C). This BR deficiency effect had been interpreted as a de-etiolated phenotype in the dark for *Arabidopsis *equivalent mutants [54].

#### Primary root elongation

Both reduced and enhanced root elongation were observed in *dgt *and *not *mutants, respectively (Figure 3D). These results are consistent with the suggestion that root growth can be regulated by a balance between auxin and ABA hormone classes [55]. Interestingly, the comparison of root elongation in the light or in the dark showed that root behavior is consistently opposite to the hypocotyl, *i.e*., primary root elongation is stimulated by light (Figure 3D). This effect could be due to a direct photomorphogenic effect, a source/sink effect, or a combination of both [56]. Whatever the correct explanation, the observed behavior favors light avoidance in tomato roots, the opposite and complementary response to dark avoidance in stems.

#### Stem height and dry weight

**Figure 4:**
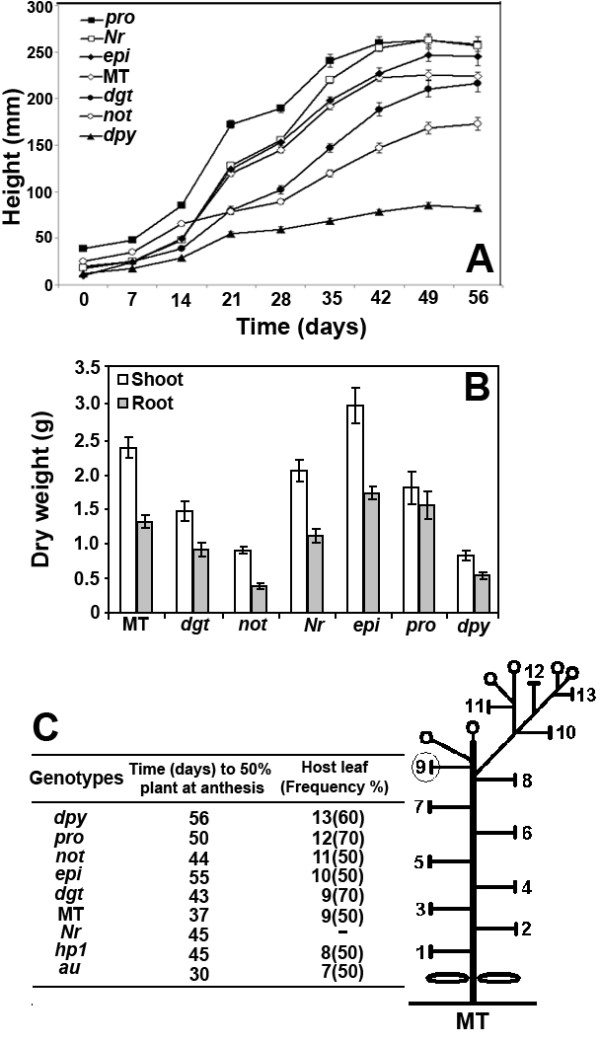
**Growth of hormone mutants in the MT background under greenhouse conditions**. (A) Growth curves of hormone mutants. (B) Dry weight of shoots (open bars) and roots (closed bars) in 60-day-old plants (n = 7). (C) Schematic representation of a 60-day-old MT plant growing in a 100-mL pot. T-shaped bars represent leaves with five leaflets (except for leaves 1 and 2, which usually have three leaflets) and small circles represent inflorescences with 5-8 flowers. Leaf 9 was highlighted for being the most frequent host of the first inflorescence in MT. This varies between mutants, the most frequent position is shown in the adjacent table with the frequency between brackets. This parameter, as well as time to anthesis in 50% of plants is indicative of developmental alterations that could increase or decrease vegetative development, in opposition to flowering. Note that MT has determinate growth (formation of two consecutive inflorescences) due to the presence of the *self-pruning *(*sp*) mutation. None of the introgressed mutants altered this trait in the MT background.

Hormone mutants presented significant differences in stem height and dry mass accumulation after 56 days of growth in greenhouse conditions (Figures 4A, 4B). Consistent with the roles of GA and auxin in cell division and expansion [57,58], GA-constitutive plants (*pro*) were considerably taller than the MT control (Figure 4A), whereas mutants with reduced sensitivity to auxin (*dgt*) showed decreased dry weight in both shoots and roots (Figure 4B). Both stem height and dry mass were reduced in ABA and BR deficient plants (*not *and *dpy*, respectively). In ABA-deficient mutants this effect can be accounted for by a generally reduced plant turgor, which leads to wilting (Figure 1O), whereas an increased endogenous ethylene level had also been proposed as the cause of the stunted growth in these mutants [59]. The observation that neither stem height nor dry mass was reduced in the ethylene overproducer mutant *epi *as in the ABA-deficient mutant (Figure 4B) suggests the existence of more components in the ABA-ethylene interaction. In agreement with that, while exogenous ABA application [59] or grafting an ABA-deficient mutant onto wild-type rootstock [60] normalized ethylene production in these mutants, their growth was still less than wild-type plants, suggesting that wild-type ABA levels are optimal for growth. The increased dry weight of *epi *also points to ethylene having a complex dual role, which can be either stimulating [61] or inhibiting [62] growth.

#### Flowering and fruit production

A series of parameters related to reproductive development were analyzed in the introgressed hormone and photomorphogenic mutants (Figure 4C and Table 3). Under our growth conditions, all mutants (except *au*) showed delayed anthesis when compared to MT (Figure 4C). This effect could be due to slower or more extended vegetative growth, which is evidenced as an increased number of leaves (or nodes) before the production of the first inflorescence. With the exception of *dgt *and *hp1*, all mutants with a delayed anthesis had an extended vegetative phase, with 10 to 13 leaves at the time of flowering, versus nine for MT (Figure 4C). BR deficiency (*dpy*), GA constitutive action (*pro*), ABA deficiency (*not*), and ethylene excess (*epi*) all extended vegetative growth. These results suggest a role for these hormones in the transition between vegetative and reproductive growth in tomato. The phytochrome deficient mutant *au *reached anthesis one week before MT, with a consistently reduced number of leaves at the onset of flowering. This points to an important role of phytochrome in tomato flowering, even when this species is considered to have little response to photoperiod [63].

**Table 3:** Parameters analyzed in flowers and fruits of hormone and photomorphogenic mutants in cv. Micro-Tom

	Flowers per inflorescence (n = 10)	Locules per fruit (frequency, n = 10)	Seeds per fruit (n = 10)	Seed weight (mg, n = 10)	Fruit weight (g, n = 10)	Total fruit weight per plant (g, n = 15)	Time (days) to fruit ripening since anthesis (n = 15)	TSS1 (n = 12)	TSS2 (n = 12)	TSS3 (n = 12)
MT	7.2 ± 0.6	3(70%)	41.3 ± 5.5	2.3 ± 0.04	5.1 ± 0.3	43.4 ± 1.9	53.4 ± 1,3	5.3 ± 0.1	5.1 ± 0.0	4.6 ± 0.1
*dgt*	8.5 ± 0.9	2(55%)	36.5 ± 4.6	2.2 ± 0.02 ^L^	3.4 ± 0.3 ^L^	28.6 ± 1.8 ^L^	57.2 ± 1,4	5.8 ± 0.2 ^H^	5.6 ± 0.2 ^H^	-
*not*	7.1 ± 0.5	2(70%)	20.4 ± 2.0 ^L^	2.5 ± 0.03 ^H^	3.7 ± 0.2 ^L^	22.2 ± 2.6 ^L^	56 ± 0,7	6.0 ± 0.1 ^H^	6.0 ± 0.1 ^H^	-
*epi*	7.2 ± 0.9	2(70%)	26.3 ± 3.9 ^L^	3.0 ± 0.02 ^H^	11.8 ± 0.5 ^H^	33.6 ± 4.8	42.1 ± 1,0 ^L^	-	4.6 ± 0.1 ^L^	-
*pro*^*a*^	4.6 ± 0.4^L^	4(85%)^H^	-	-	3.4 ± 0.2 ^L^	45.4 ± 2.5	54.8 ± 2,3	9.1 ± 0.5 ^H^	7.5 ± 0.5 ^H^	-
*pro*^*b*^	-	-	-	2.7 ± 0.03 ^H^	-	-	-	8.1 ± 0.2 ^H^	-	-
*gib3*	-	-	-	2.0 ± 0.03 ^L^	-	-	-	-	-	-
*dpy*	6.1 ± 0.2	2(70%)	22.4 ± 3.8 ^L^	2.3 ± 0.01	4.0 ± 0.4 ^L^	12.4 ± 1.5 ^L^	58.7 ± 2,6	4.9 ± 0.0 ^L^	4.8 ± 0.1 ^L^	-
*au*	10.8 ± 0,5^H^	2 (70%)	41 ± 2.1	2.2 ± 0.01 ^L^	5.1 ± 0.3	40 ± 0.8	48.6 ± 1.0 ^L^	-	-	5.0 ± 0.1 ^H^
*hp1*	8 ± 0,6	2 (80%)	31.1 ± 1.8	2.7 ± 0.02 ^H^	9.4 ± 0.4 ^H^	44.5 ± 1.1	58 ± 2.0	-	-	5.4 ± 0.1 ^H^

^a ^Partenocarpic fruit, ^b ^Fruit derived from artificial pollination, TSS1 = Total Soluble Solids (Brix) measured in winter 2005, TSS2 = measured in summer 2006, TSS3 = measured in autumn 2007. Mean values are followed by standard error (see Methods for experiment design). The letters H and L represent values statistically significantly higher or lower than MT, respectively (P < 0.05, Student *t*-test).

Various agronomically important responses were assessed on the mutants from anthesis to fruit ripening (Table 3). The most relevant observations are summarized here: i) increase and decrease in number of flowers per inflorescence in *au *and *pro*, respectively; ii) a tendency in most mutants to develop two locules per fruit instead of three as MT, as well as supernumerary locules in *pro*; iii) expressive decrease in seed number per fruit in *not*, *epi *and *dpy*; iv) seed weight increase in *epi*, *pro*, *hp1*, and *not *and decrease in *gib3*, *dgt *and *au*; v) increase of fruit weight in *epi *and *hp1 *and decrease in all other mutants, except *au *(which showed no change); vi) decreased yield (total fruit weight per plant) in *dgt*, *not *and *dpy*; vii) decrease in ripening time in *epi *and *au*; viii) increased total soluble solids (TSS) in most mutants, mainly in *pro*, and decreased values in *epi *and *dpy*.

In agronomic terms, the most significant result was the observation that parthenocarpic as well as normally pollinated fruit of *pro *mutant showed increased TSS (Table 3). The *pro *mutation also led to a reduction in fruit weight but not in yield, whereas the gain in TSS was in excess of 60%. Wild species of the former *Lycopersicon *genus can produce as much as double the TSS as tomato. However, inheritance of this trait is polygenic and the highest increase gained with major genes derived from wild species reported so far has been 20% [64,65].

## Discussion

**Figure 5:**
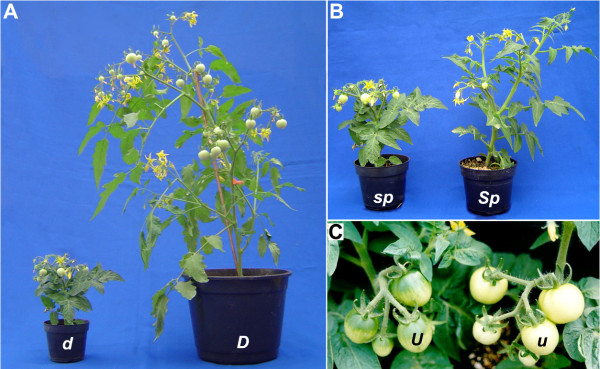
**Lines near isogenic to MT harboring wild type alleles for *DWARF *(*D*), *SELF-PRUNING *(*SP*) and *UNIFORM RIPENING *(*U*) genes**. (A) MT plant harboring the *D *allele still shows a reduced size (as compared to common cultivars), but does not show rough leaves and is significantly bigger than MT harboring the *d *allele. (B) The wild *Sp *allele in the MT background produces taller plants due to the extended growth of its vegetative apices, which denotes the indeterminate growth habit in opposition to the determinate one conferred by *sp*. The plants shown are of same age. Note the presence of leaves between inflorescences in *Sp*. (C) Plants harboring the *U *allele develop fruits with a green shoulder. The pale bright color of MT fruits is probably a result not only of the *u *mutation, but also *uniform gray-green *(*ug*).

In this work we present a collection of tomato mutations introgressed into the MT cultivar. Various hormone and photomorphogenetic mutants were introduced into this single genetic background in a short period of time and within limited growth facilities. This is a less labor-intensive approach than induced mutagenesis, which would require considerable infrastructure to screen a large amount of plants. Further, for some loci, particularly those that are already knocked-out in the chosen model, the introgression of functional alleles may be the only way to restore the phenotypic variation (*i.e*. the gene effect) in order to study gene function [66]. As proof of concept, we quickly introgressed into MT the wild type alleles *Dwarf *(*D *- Figure 5A), *Self-pruning *(*Sp *- Figure 5B) and *Uniform ripening *(*U *- Figure 5C). MT itself harbors non-functional alleles of all these genes, which are responsible for the small size (*d *and *sp*), plant determinate growth (*sp) *and uniform fruit color (*u*) of the cultivar [4,13]. Serrani *et al*. [9] recently used MT to assess crosstalk between GA and auxin in the formation of parthenocarpic fruits in tomato. The authors also used MT *D *and MT *Sp *as controls to MT itself and showed that, although MT harbors mutated versions of both genes (*d *and *sp*), no difference was observed in the evaluated responses between MT, MT *D *and MT *Sp*. Further, since tomato is often used in grafting experiments [60], the longer stem of MT *D *line has an obvious advantage in the grafting procedure.

In the newly introgressed genotypes, the reduced size of MT proved to be additive to all the hallmark phenotypes of either hormonal or photomorphogenetic mutations. Among them stand out the extreme dwarfism of GA-deficient (*gib1*, *gib2 *and *gib3*) and BR insensitive/deficient mutants (*cu3 *and *dpy *respectively) in the MT background. As already known, the dwarfism in MT is partially due to two recessive mutations, one of which is allelic to the aforementioned *dwarf *[13,67], a gene in the BR biosynthesis pathway [68]. It follows from this that MT has reduced levels of BR, but without the dramatic effects evident in the extreme *dwarf *allele *d*^*x *^[68], which resembles *cu3 *and *dpy *[23]. The additive phenotype of *cu3 *and *dpy *in MT suggests that the second mutation conferring dwarfism to MT is not related to BR. Thus, the presence of a second mutation affecting BR in MT would already result in a phenotype similar to *dpy *and *cu3*, as a consequence of the amplified effect of stacking mutant alleles of genes from the same pathway (see for instance Figure 2K). A further indication of this is the absence of rough leaves, a typical BR-deficiency phenotype, in MT plants lacking the *d *allele but still harboring the second mutation conferring dwarfism (MT-*D*, Figure 5A). The reduction in size produced by GA-deficiency mutations in MT (Figure 1M), as well as the phenotype produced by the GA-constitutive mutation *pro *(Figure 1K) are in agreement with the previous suggestion that MT's dwarfism is not fully explained by GA effects either [13]. More conclusively, in contrast to every known GA-deficient mutant in tomato [22], MT shows a seed germination rate considered normal for a wild-type genotype (Figures 3A, 3B). Taken together, these results suggest that, in spite of being a mild BR mutant, MT and the collection itself are perfectly suitable tools for understanding plant hormone interactions. Moreover, if the event under study is influenced by *dwarf*, or even any other mutation that MT already holds, the alternative of generating near isogenic lines (NILs) harboring the non-mutated allele as a control, such as MT *D*, MT *Sp *and MT *U *presented here (Figure 5), fulfills the requirement of an appropriate control in the scientific method. Thus, all mutants obtained in MT by introgression (Table 1 and 2) or mutagenesis [13,67] can be now easily combined with the *D*, *S*p and *U *wild type alleles, as double mutants, when necessary.

Some of the alleles presented in the collection are not derived from other cultivars, but from related species within the *Solanum *section *Lycopersicum*. *Ip *and *atv *(Figure 2; Table 2) were introgressed from *Solanum chmielewskii *and *S. cheesmaniae *respectively, and *cu3 *from *S. pimpinellifolium *[24]. We have also previously described the introgression of *Rg1 *into MT [67], which increases *in vitro *regeneration capacity and was originally found in *S. peruvianum *[69]. The feasibility of comparative studies between alleles from related wild species in the MT background also makes the collection amenable for the study of natural genetic variation [66,70]. Combining natural genetic variation and other mutations in a single and more tractable genetic background will improve the capacity to observe novel phenotypes and gene interactions (epistasis). Most of the novel phenotypes reported here for previously-studied mutants is probably the consequence of such improvement in the capacity of observation, since they are very coherent with the function of the altered pathway. This seems to be the case of the presence of navel-like (and parthenocarpic) fruits, precocious germination and high brix in the GA-constitutive *pro *mutant; or the early flowering and fruit ripening of the phytochrome-mutant *au*. However, we could not exclude the occurrence of some epistatic interactions with other mutations already present in the MT background, which may produce novel phenotypes such as the high dry weight of shoot and fruit observed in the ethylene overproducer *epi *as opposed to its original description [16].

Finally, various mutants from the collection presented here have already been used to investigate a wide range of topics in plant biology. Among them is the role of hormone classes in the light-induced anthocyanin accumulation in tomato hypocotyls [71], the multi-hormonal control of defense against herbivory in tomato [8], the role of plant hormones in the development of arbuscular mycorrhizae in tomato roots [72] and callus, shoot, and hairy-root formation in hormonal mutants [73]. Taken together, the present work is good evidence that the present collection of tomato mutants in a single genetic background is a suitable approach to conduct research in plant development.

## Methods

### Breeding and cultivation

*Solanum lycopersicum *L. cv. Micro-Tom (MT) plants were grown in a greenhouse with automatic irrigation (four times/day to field capacity), mean temperature of 28°C, 11.5 h/13 h (winter/summer) photoperiod, and 250 to 350 μmol photons m^-2 ^sec^-1 ^PAR irradiance, attained by reduction of natural radiation with a reflecting mesh (Aluminet, Polysack Industrias Ltda, Leme, Brazil). Mutant seeds were germinated in trays containing a 1:1 mixture of commercial mix (Plantmax HT, Eucatex, Brazil) and expanded vermiculite, supplemented with 1 g L^-1 ^10:10:10 NPK and 4 g L^-1 ^lime. Ten days after germination, plants were transferred to 150 mL (MT) or 10 L (other cultivars/species) pots containing soil mix. After crossing, mature fruits were harvested and the pulp removed from the seed by inoculation and overnight fermentation with *Saccharomyces cerevisae *(Fermix, Brazil). Seeds were further washed and air-dried in preparation for germination.

The mutations of interest were introgressed into the MT cultivar by a series of crosses and back-crosses (see Figure S1). Pollen was collected from parent plants and used to fertilize emasculated MT flowers (floral organs were ready for emasculation 35 days after sowing). The resulting F_1 _hybrids were selfed to obtain recombinant F_2 _populations, which were subsequently screened for compact size and the mutation of interest. The selected plants were backcrossed with MT up to the sixth generation (BC_6_), selfing every second generation to screen for homozygous mutants. After BC_6_F_2 _the resulting genotypes can be considered near-isogenic lines [74]. For dominant mutations (e.g., *Never ripe: Nr*), selfing was skipped until the BC_6 _generation, when BC_6_F_2 _homozygous plants were identified through observation of their derived seedlings (BC_6_F_3_). *Nr *fruit was harvested unripe (since the mutation impairs normal ripening) and the ABA-deficient mutants *sitiens *(*sit*), *flacca *(*flc*) and *notabilis *(*not*) were harvested prematurely in order to avoid seed germination during fermentation. GA-deficient mutants (*gib1, 2 and 3*) were screened by germinating seeds onto wet filter paper. GA-deficient mutants do not normally germinate without exogenous GA application. After one week, germinating seeds were discarded, and the remaining batch was transferred to boxes containing filter paper soaked with 100 μM GA_3_. The seeds that germinated after one week of GA treatment were transferred to the greenhouse where the mutant phenotype (Figure 1M) was confirmed. The GA-deficiency phenotype was only observable when residual exogenous GA effects were negligible, which normally occurred two weeks after transferring the seedlings to the greenhouse. After this screening, 100 μM GA_3 _was sprayed fortnightly to allow flowering and fruit set [22].

### Seed germination and seedling measurements

Assays on germination time and hypocotyl and root length of dark-grown seedlings were performed by sowing seeds onto wet filter paper in black plastic boxes. Time to germination (seeds with visible radicle) was assessed daily over five days in three replicate experiments (150 seeds per treatment). Seeds were counted in a dark room under green light. Hypocotyl and root lengths were measured after 10 days in 20 seedlings per treatment. For light treatments, seed germination was assessed in growth cabinets (Marconi, Piracicaba, Brazil) *in vitro *under light in a growth chamber (25°C, 16 h photoperiod, 55 μmol photons m^-2 ^sec^-1 ^PAR). Seed batches had been harvested at the same time from plants grown under the same conditions.

### Evaluation of vegetative and reproductive traits

Plant height was measured weekly over 56 days in 15 plants per treatment. Stems and roots of 60-day-old plants (n = 7) were oven-dried at 60°C and their dry mass determined. Number of flowers was recorded in 15 plants starting from the first complete inflorescence (n = 15). Time from anthesis to ripe fruit was measured in 15 fruits attached to different mother plants. Seeds per fruit, fruit weight and locule number per fruit were determined on 10 fruits per treatment (n = 10). Seed weight was determined for 100 seeds in 10 replicates (n = 10). Total fruit weight per plant (yield) was measured in 15 plants per treatment (n = 15). Total soluble solids (TSS) were measured in the flesh of ripe fruit using a digital refractometer (Atago PR-101) on 12 fruits per treatment (n = 12) derived from 12 different plants.

## Competing interests

The authors declare that they have no competing interests.

## Authors' contributions

MLC, RFC, LEP, SLC, AZ, and JEL performed crosses and characterized specific mutants into MT background. VAB and MLC participated in discussions, on mutant characterization, and worked on manuscript preparation. LEPP conceived the project, designed the introgressions, performed some backcrosses and worked on manuscript preparation. All authors read and approved the final manuscript.

## Supplementary Material

Additional file 1 **Figure S1**. Scheme of the backcross introgression process. Click here for file

## References

[B1] Sol Genomics Network http://solgenomics.net

[B2] The C. M Rick Tomato Genetics Resource Center http://tgrc.ucdavis.edu

[B3] Pino LE Lombardi-Crestana S Azevedo MS Scotton DC Borgo L Quecini V Figueira A Peres LEP The *Rg1 *allele as a valuable tool for genetic transformation of the tomato Micro-Tom model system Plant Methods 2010 6 23 10.1186/1746-4811-6-23 20929550 PMC2958934

[B4] Campos ML Carvalho RF Benedito VA Peres LEP Small and remarkable: the Micro-Tom model system as a tool to discover novel hormonal functions and interactions Plant Signal Behav 2010 5 50 54 10.4161/psb.5.3.10622 PMC2881274 20037476

[B5] Scott J Harbaugh B Micro-Tom: A miniature dwarf tomato Florida Agric Exp Station Circular 1989 370 1 6

[B6] Meissner R Jacobson Y Melamed S Levyatuv S Shalev G Ashri A Elkind Y Levy A A new model system for tomato genetics Plant J 1997 12 1465 1472 10.1046/j.1365-313x.1997.12061465.x

[B7] Wang H Schauer N Usadel B Frasse P Zouine M Hernould M Latche A Pech JC Fernie AR Bouzayen M Regulatory features underlying pollination-dependent and -independent tomato fruit set revealed by transcript and primary metabolite profiling Plant Cell 2009 21 1428 1452 10.1105/tpc.108.060830 19435935 PMC2700536

[B8] Campos ML Almeida M Rossi ML Martinelli AP Junior CGL Figueira A Rampelotti-Ferreira FT Vendramim JD Benedito VA Peres LEP Brassinosteroids interact negatively with jasmonates in the formation of anti-herbivory traits in tomato J Exp Bot 2009 60 4347 4361 10.1093/jxb/erp270 19734261

[B9] Serrani JC Carrera E Ruiz-Rivero O Gallego-Giraldo L Peres LEP Garcia-Martinez JL Inhibition of auxin transport from the ovary or from the apical shoot induces parthenocarpic fruit-set in tomato mediated by gibberellins Plant Physiol 2010 153 851 862 10.1104/pp.110.155424 20388661 PMC2879769

[B10] Emmanuel E Levy AA Tomato mutants as tools for functional genomics Curr Opin Plant Biol 2002 5 112 117 10.1016/S1369-5266(02)00237-6 11856605

[B11] Tonsor SJ Alonso-Blanco C Koornneef M Gene function beyond the single trait: natural variation, gene effects, and evolutionary ecology in *Arabidopsis thaliana* Plant Cell Environ 2005 28 2 20 10.1111/j.1365-3040.2004.01264.x

[B12] Dowell RD Ryan O Jansen A Cheung D Agarwala S Danford T Bernstein DA Rolfe A Heisler LE Chin B Nislow C Giaever G Phillipis PC Fink GR Gifford DK Boone C Genotype to phenotype: a complex problem Science 2010 328 469 10.1126/science.1189015 20413493 PMC4412269

[B13] Martí E Gisbert C Bishop GJ Dixon MS Garcia-Martinez JL Genetic and physiological characterization of tomato cv. Micro-Tom J Exp Bot 2006 57 2037 2047 10.1093/jxb/erj154 16687436

[B14] Oh K Ivanchenko MG White TJ Lomax TL The *diageotropica *gene of tomato encodes a cyclophilin: a novel player in auxin signaling Planta 2006 224 133 144 10.1007/s00425-005-0202-z 16395583

[B15] Scott IM Effects of gibberellin on shoot development in the *dgt *mutant of tomato Ann Bot 1988 61 389 392

[B16] Fujino DW Burger DW Yang SF Bradford KJ Characterization of an ethylene overproducing mutant of tomato (*Lycopersicon esculentum *Mill cultivar VFN8) Plant Physiol 1988 88 774 779 10.1104/pp.88.3.774 16666382 PMC1055659

[B17] Wilkinson JQ Lanahan MB Yen HC Giovannoni JJ Klee HJ An ethylene-inducible component of signal-transduction encoded by *Never-ripe* Science 1995 270 1807 1809 10.1126/science.270.5243.1807 8525371

[B18] Lanahan MB Yen HC Giovannoni JJ Klee HJ The *Never ripe *mutation blocks ethylene perception in tomato Plant Cell 1994 6 521 530 8205003 10.1105/tpc.6.4.521 PMC160455

[B19] Bassel GW Mullen RT Bewley JD *procera *is a putative DELLA mutant in tomato (*Solanum lycopersicum*): effects on the seed and vegetative plant J Exp Bot 2008 59 585 593 10.1093/jxb/erm354 18250077

[B20] van Tuinen A Peters AHLJ Kendrick RE Zeevaart JAD Koornneef M Characterisation of the *procera *mutant of tomato and the interaction of gibberellins with end-of-day far-red light treatments Physiol Plant 1999 106 121 128 10.1034/j.1399-3054.1999.106117.x

[B21] Bensen RJ Zeevaart JAD Comparison of *ent*-kaurene synthetase A-activity and B-activity in cell-free-extracts from young tomato fruits of wild-type and *gib-1*, *gib-2*, and *gib-3 *tomato plants J Plant Growth Regul 1990 9 237 242

[B22] Koornneef M Bosma TDG Hanhart CJ Vanderveen JH Zeevaart JAD The isolation and characterization of gibberellin-deficient mutants in tomato Theor Appl Genet 1990 80 852 857 10.1007/BF00224204 24221121

[B23] Koka CV Cerny RE Gardner RG Noguchi T Fujioka S Takatsuto S Yoshida S Clouse SD A putative role for the tomato genes *DUMPY *and *CURL-3 *in brassinosteroid biosynthesis and response Plant Physiol 2000 122 85 98 10.1104/pp.122.1.85 10631252 PMC58847

[B24] Montoya T Nomura T Farrar K Kaneta T Yokota T Bishop GJ Cloning the tomato *curl3 *gene highlights the putative dual role of the leucine-rich repeat receptor kinase tBRI1/SR160 in plant steroid hormone and peptide hormone signaling Plant Cell 2002 14 3163 3176 10.1105/tpc.006379 12468734 PMC151209

[B25] Zeevaart J Hooykaas P, Hall M, Libbenga K Abscisic acid metabolism and its regulation Biochemistry and Molecular Biology of Plant Hormones 1999 Amsterdam: Elsevier Science 189 207

[B26] Taylor IB Burbidge A Thompson AJ Control of abscisic acid synthesis J Exp Bot 2000 51 1563 1574 10.1093/jexbot/51.350.1563 11006307

[B27] Burbidge A Grieve TM Jackson A Thompson A McCarty DR Taylor IB Characterization of the ABA-deficient tomato mutant *notabilis *and its relationship with maize *Vp14* Plant J 1999 17 427 431 10.1046/j.1365-313X.1999.00386.x 10205899

[B28] Frey A Audran C Marin E Sotta B Marion-Poll A Engineering seed dormancy by the modification of zeaxanthin epoxidase gene expression Plant Molecular Biology 1999 39 1267 1274 10.1023/A:1006145025631 10380812

[B29] Fankhauser C Chory J Light control of plant development Annu Rev Cell Dev Biol 1997 13 203 229 10.1146/annurev.cellbio.13.1.203 9442873

[B30] Smith H Phytochromes and light signal perception by plants - an emerging synthesis Nature 2000 407 585 591 10.1038/35036500 11034200

[B31] Muramoto T Kami C Kataoka H Iwata N Linley PJ Mukougawa K Yokota A Kohchi T The tomato photomorphogenetic mutant, *aurea*, is deficient in phytochromobilin synthase for phytochrome chromophore biosynthesis Plant Cell Physiol 2005 46 661 665 10.1093/pcp/pci062 15695440

[B32] Davis SJ Bhoo SH Durski AM Walker JM Vierstra RD The heme-oxygenase family required for phytochrome chromophore biosynthesis is necessary for proper photomorphogenesis in higher plants Plant Physiol 2001 126 656 669 10.1104/pp.126.2.656 11402195 PMC111157

[B33] Koornneef M Cone JW Dekens RG O'Herne-Robers EG Spruit CJP Kendrick RE Photomorphogenic responses of long hypocotyl mutants of tomato J Plant Physiol 1985 120 153 165

[B34] van Tuinen AV Hanhart CJ Kerckhoffs LHJ Nagatani A Boylan MT Quail PH Kendrick RE Koornneef M Analysis of phytochrome-deficient *yellow-green-2 *and *aurea *mutants of tomato Plant J 1996 9 173 182 10.1046/j.1365-313X.1996.09020173.x

[B35] Liu YS Roof S Ye ZB Barry C van Tuinen A Vrebalov J Bowler C Giovannoni J Manipulation of light signal transduction as a means of modifying fruit nutritional quality in tomato Proc Natl Acad Sci USA 2004 101 9897 9902 10.1073/pnas.0400935101 15178762 PMC470770

[B36] Mustilli AC Fenzi F Ciliento R Alfano F Bowler C Phenotype of the tomato *high pigment-2 *mutant is caused by a mutation in the tomato homolog of *DEETIOLATED1* Plant Cell 1999 11 145 157 9927635 10.1105/tpc.11.2.145 PMC144164

[B37] Mochizuki T Kamimura S Photoselective method for selection of *hp *at the cotyledon stage Tomato Genet Coop Rep 1985 35 12 13

[B38] Kendrick RE Kerckhoffs LHJ van Tuinen A Koornneef M Photomorphogenic mutants of tomato Plant Cell Environ 1997 20 746 751 10.1046/j.1365-3040.1997.d01-109.x

[B39] Rick CM Reeves AF Zobel RW Inheritance and linkage relations of four new mutants Tomato Genet Coop Rep 1968 18 34 35

[B40] Rick CM High soluble-solids content in large-fruited tomato lines derived from a wild green-fruited species Hilgardia 1974 42 493 510

[B41] Kucera B Cohn MA Leubner-Metzger G Plant hormone interactions during seed dormancy release and germination Seed Sci Res 2005 15 281 307 10.1079/SSR2005218

[B42] Brady SM McCourt P Hormone cross-talk in seed dormancy J Plant Growth Regul 2003 22 25 31 10.1007/s00344-003-0018-7

[B43] Siriwitayawan G Geneve RL Downie AB Seed germination of ethylene perception mutants of tomato and Arabidopsis Seed Sci Res 2003 13 303 314 10.1079/SSR2003147

[B44] Chiwocha SDS Cutler AJ Abrams SR Ambrose SJ Yang J Ross ARS Kermode AR The *etr1-2 *mutation in *Arabidopsis thaliana *affects the abscisic acid, auxin, cytokinin and gibberellin metabolic pathways during maintenance of seed dormancy, moist-chilling and germination Plant J 2005 32 35 48 10.1111/j.1365-313X.2005.02359.x 15773852

[B45] Steber CM McCourt P A role for brassinosteroids in germination in Arabidopsis Plant Physiol 2001 125 763 769 10.1104/pp.125.2.763 11161033 PMC64877

[B46] Georghiou K Kendrick RE The germination characteristics of phytochrome-deficient *aurea *mutant tomato seeds Physiol Plant 1991 82 127 133 10.1111/j.1399-3054.1991.tb02912.x

[B47] Kerckhoffs LHJ Schreuder MEL van Tuinen A Koornneef M Kendrick RE Phytochrome control of anthocyanin biosynthesis in tomato seedlings: analysis using photomorphogenic mutants Photochem Photobiol 1997 65 374 381 10.1111/j.1751-1097.1997.tb08573.x

[B48] Alabadí D Blázquez MA Molecular interactions between light and hormone signaling to control plant growth Plant Mol Biol 2009 69 409 417 10.1007/s11103-008-9400-y 18797998

[B49] Halliday KJ Fankhauser C Phytochrome-hormonal signalling networks New Phytol 2003 157 449 463 10.1046/j.1469-8137.2003.00689.x 33873406

[B50] Kim BC Soh MS Kang BJ Furuya M Nam HG Two dominant photomorphogenic mutations of *Arabidopsis thaliana *identified as suppressor mutations of *hy2* Plant J 1996 9 441 456 10.1046/j.1365-313X.1996.09040441.x 8624510

[B51] Kraepiel Y Agnes C Thiery L Maldiney R Miginiac E Delarue M The growth of tomato (*Lycopersicon esculentum *Mill.) hypocotyls in the light and in darkness differentially involves auxin Plant Sci 2001 161 1067 1074 10.1016/S0168-9452(01)00495-2 12088031

[B52] Collett CE Harberd NP Leyser O Hormonal interactions in the control of Arabidopsis hypocotyl elongation Plant Physiol 2000 124 553 561 10.1104/pp.124.2.553 11027706 PMC59162

[B53] Barry CS Fox EA Yen HC Lee S Ying TJ Grierson D Giovannoni JJ Analysis of the ethylene response in the *epinastic *mutant of tomato Plant Physiol 2001 127 58 66 10.1104/pp.127.1.58 11553734 PMC117962

[B54] Li JM Chory J A putative leucine-rich repeat receptor kinase involved in brassinosteroid signal transduction Cell 1997 90 929 938 10.1016/S0092-8674(00)80357-8 9298904

[B55] Peres LEP Zsögön A Kerbauy GB Abscisic acid and auxin accumulation in *Catasetum fimbriatum *roots growing *in vitro *with high sucrose and mannitol content Biol Plantarum 2009 53 560 564 10.1007/s10535-009-0101-4

[B56] Drozdova IS Bondar VV Bukhov NG Kotov AA Kotova LM Maevskaya SN Mokronosov AT Effects of light spectral quality on morphogenesis and source-sink relations in radish plants Russ J Plant Physiol 2001 48 415 420 10.1023/A:1016725207990

[B57] Jones AM Im KH Savka MA Wu MJ DeWitt NG Shillito R Binns AN Auxin-dependent cell expansion mediated by overexpressed auxin-binding protein 1 Science 1998 282 1114 1117 9804548 10.1126/science.282.5391.1114

[B58] Jones MG Gibberellins and the *procera *mutant of tomato Planta 1987 172 280 284 10.1007/BF00394598 24225881

[B59] Sharp RE LeNoble ME Else MA Thorne ET Gherardi F Endogenous ABA maintains shoot growth in tomato independently of effects on plant water balance: evidence for an interaction with ethylene J Exp Bot 2000 51 1575 1584 10.1093/jexbot/51.350.1575 11006308

[B60] Dodd IC Theobald JC Richer SC Davies WJ Partial phenotypic reversion of ABA-deficient *flacca *tomato (*Solanum lycopersicum*) scions by a wild-type rootstock: normalizing shoot ethylene relations promotes leaf area but does not diminish whole plant transpiration rate J Exp Bot 2009 60 4029 4039 10.1093/jxb/erp236 19648172 PMC2755025

[B61] Satler SO Kende H Ethylene and the growth of rice seedlings Plant Physiol 1985 79 194 198 10.1104/pp.79.1.194 16664369 PMC1074850

[B62] Abeles FB Saltveit ME Jr Ethylene in Plant Biology 1992 2 San Diego: Academic Press

[B63] Lifschitz E Eviatar T Rozman A Goldshmidt A Amsellem Z Alvarez JP Esched Y The tomato *FT *ortholog triggers systemic signals that regulate growth and flowering and substitute for diverse environmental stimuli Proc Natl Acad Sci USA 2006 103 6398 6403 10.1073/pnas.0601620103 16606827 PMC1458889

[B64] Fridman E Carrari F Liu YS Fernie AR Zamir D Zooming in on a quantitative trait for tomato yield using interspecific introgressions Science 2004 305 1786 1789 10.1126/science.1101666 15375271

[B65] Taylor IB Atherton JG, Rudich J Biosystematics of the tomato The Tomato Crop: a scientific basis for improvement 1986 London: Chapman and Hall 1 34

[B66] Alonso-Blanco C Koornneef M Naturally occurring variation in Arabidopsis: an underexploited resource for plant genetics Trends Plant Sci 2000 5 22 29 10.1016/S1360-1385(99)01510-1 10637658

[B67] Lima JE Carvalho RF Neto AT Figueira A Peres LEP Micro-MsK: a tomato genotype with miniature size, short life cycle, and improved *in vitro *shoot regeneration Plant Sci 2004 167 753 757 10.1016/j.plantsci.2004.05.023

[B68] Bishop GJ Nomura T Yokota T Harrison K Noguchi T Fujioka S Takatsuto S Jones JDG Kamiya Y The tomato DWARF enzyme catalyses C-6 oxidation in brassinosteroid biosynthesis Proc Natl Acad Sci USA 1999 96 1761 1766 10.1073/pnas.96.4.1761 9990098 PMC15587

[B69] Koornneef M Bade J Hanhart C Horsman K Schel J Soppe W Verkerk R Zabel P Characterization and mapping of a gene controlling shoot regeneration in tomato Plant J 1993 3 131 141 10.1111/j.1365-313X.1993.tb00016.x

[B70] Rick CM Hollaender A, Srb AM Potential genetic resources in tomato species: clues from observations in native habitats Genes, Enzymes and Populations 1973 New York: Plenum Press 255 269 10.1007/978-1-4684-2880-3_17 4792359

[B71] Carvalho RF Quecini V Peres LEP Hormonal modulation of photomorphogenesis-controlled anthocyanin accumulation in tomato (*Solanum lycopersicum *L. cv. Micro-Tom) hypocotyls: physiological and genetic studies Plant Sci 2010 178 258 264 10.1016/j.plantsci.2010.01.013

[B72] Zsögön A Lambais MA Benedito VA Figueira AVO Peres LEP Reduced arbuscular mycorrhizal colonization in tomato ethylene mutants Sci Agr 2008 65 259 267

[B73] Lima JE Benedito VA Figueira A Peres LEP Callus, shoot and hairy root formation *in vitro *is affected by the sensitivity to auxin and ethylene in tomato mutants Plant Cell Rep 2009 28 1169 1177 10.1007/s00299-009-0718-y 19484241

[B74] Reid JB Plant hormone mutants J Plant Growth Reg 1993 12 207 226

