# Supplementary material for: Convergence of developmental mutants into a single tomato model system: 'Micro-Tom' as an effective toolkit for plant development research
Source: Plant Methods. 2011 Jun 29;7:18. doi: 10.1186/1746-4811-7-18 (PMC3146949; doi:10.1186/1746-4811-7-18)
Supplement: Additional file 1 — Figure S1. Scheme of the backcross introgression process. [file 1746-4811-7-18-S1.PDF]

Convergence of developmental mutants into a single tomato model system: ‘Micro-Tom’ as an effective toolkit for plant development research

Rogério Falleiros Carvalho, Marcelo Lattarulo Campos, Lilian Ellen Pino, Simone Lombardi Crestana, Agustin Zsögön, Joni Esrom Lima, Vagner Augusto Benedito and Lázaro Eustáquio Pereira Peres

Supplementary Material

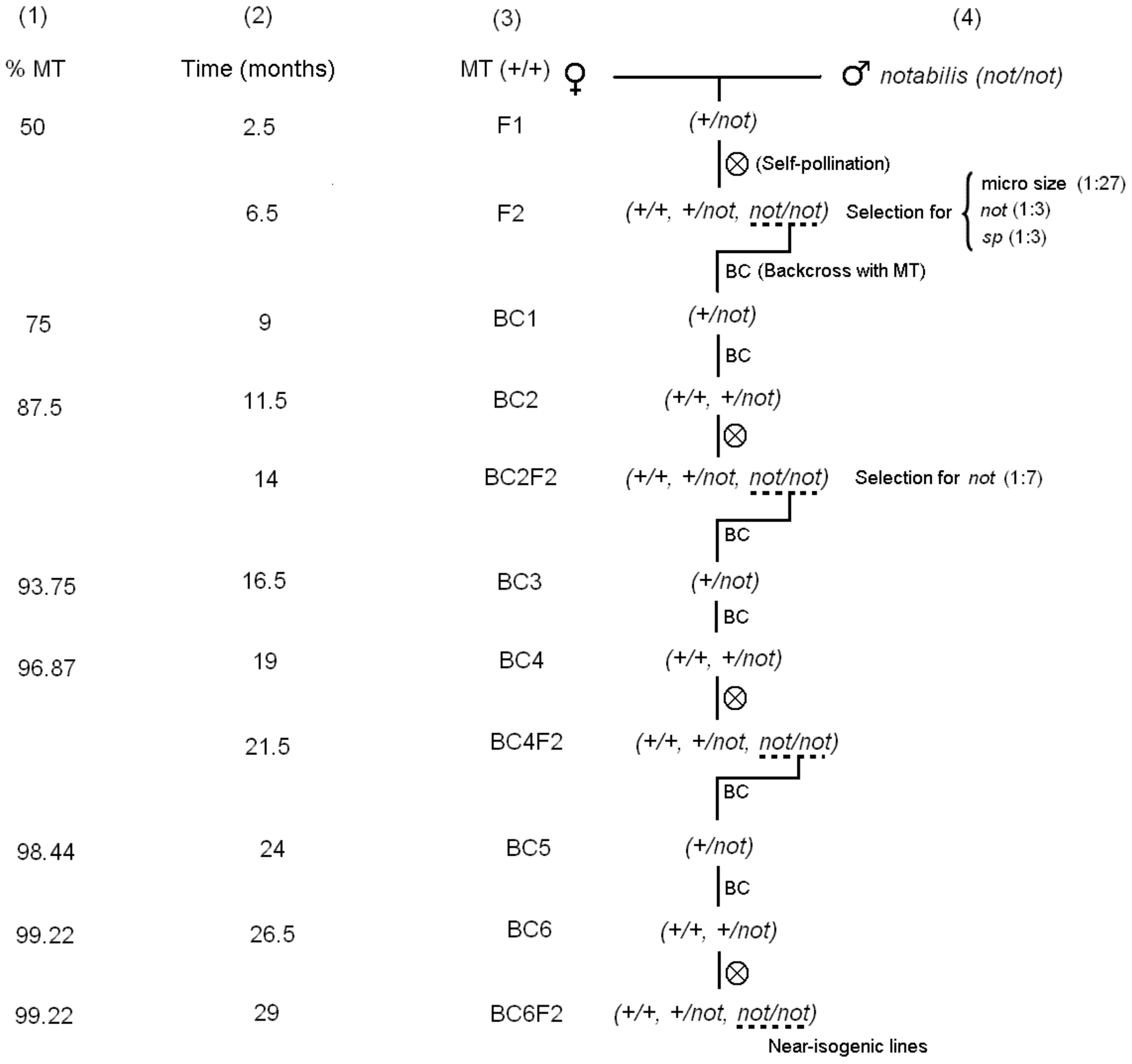

Fig. S1. Scheme of the introgression process. Hormone mutants, represented here by *notabilis* (*not*), a recessive mutation, were introgressed into the Micro-Tom (MT) background through a series of six backcrosses (BCs) and selfings (BCnF2). (1) Theoretical proportion of the MT genome in each generation. (2) Time (months) to reach each generation. (3) Nomenclature of each generation representing crosses and backcrosses (F1 and BCn) and selfings (F2, BCnF2). Selfings were necessary to visualize the phenotype of recessive mutations (e.g. *not*), which extended the introgression period through 29 months. In the case of dominant mutations (e.g. *Nr*), the process would take around 24 months. In F2, seeds were sown in high density and 14-day-old seedlings were selected for MT size, with a ratio of 1:27 (3.5%). In the resulting plants, the screening was for wilty plants (*not*/*not*) and subsequently, for determinate growth (*sp*/*sp*), with a ratio of 1:3 (25%) in each screening. Thus, the final ratio of selected plants in F2 is 1:447 (0.2%). In subsequent BCs, the dwarf habit and determined growth stay fixed, there being no further need for selection, since the recurrent parental (MT) also harbors those traits. The remaining of the introgression process is the selection of *not* in BC2F2, BC4F2 and BC6F2, where the ratio was 1:7 (12.5%).
